# Fungal Alcohol Dehydrogenases: Physiological Function, Molecular Properties, Regulation of Their Production, and Biotechnological Potential

**DOI:** 10.3390/cells12182239

**Published:** 2023-09-08

**Authors:** J. Félix Gutiérrez-Corona, Gloria Angélica González-Hernández, Israel Enrique Padilla-Guerrero, Vianey Olmedo-Monfil, Ana Lilia Martínez-Rocha, J. Alberto Patiño-Medina, Víctor Meza-Carmen, Juan Carlos Torres-Guzmán

**Affiliations:** 1 Departamento de Biología, DCNE, Universidad de Guanajuato, Guanajuato C.P. 36050, Mexico; gonzang@ugto.mx (G.A.G.-H.); ie.padillaguerrero@ugto.mx (I.E.P.-G.); vg.olmedo@ugto.mx (V.O.-M.); anamartinez@ugto.mx (A.L.M.-R.); 2 Instituto de Investigaciones Químico Biológicas, Universidad Michoacana de San Nicolás de Hidalgo (UMSNH), Morelia C.P. 58030, Mexico; jpatino@umich.mx (J.A.P.-M.); victor.meza@umich.mx (V.M.-C.)

**Keywords:** alcohol dehydrogenase, biological interactions, biotechnology prospective, fungi, function, transcriptional regulation, taxonomy, zinc-dependent

## Abstract

Fungal alcohol dehydrogenases (ADHs) participate in growth under aerobic or anaerobic conditions, morphogenetic processes, and pathogenesis of diverse fungal genera. These processes are associated with metabolic operation routes related to alcohol, aldehyde, and acid production. The number of ADH enzymes, their metabolic roles, and their functions vary within fungal species. The most studied ADHs are associated with ethanol metabolism, either as fermentative enzymes involved in the production of this alcohol or as oxidative enzymes necessary for the use of ethanol as a carbon source; other enzymes participate in survival under microaerobic conditions. The fast generation of data using genome sequencing provides an excellent opportunity to determine a correlation between the number of ADHs and fungal lifestyle. Therefore, this review aims to summarize the latest knowledge about the importance of ADH enzymes in the physiology and metabolism of fungal cells, as well as their structure, regulation, evolutionary relationships, and biotechnological potential.

## 1. Introduction

Alcohol dehydrogenases (ADHs) (EC 1.1.1.1) are oxidoreductases that catalyze the interconversion of alcohols, aldehydes, and ketones. Oxidoreductases have been classified into three main categories: (1) NAD- or NADP-dependent dehydrogenases; (2) NAD(P)-independent enzymes that use pyrroloquinoline quinone, haem, or F420 as a cofactor; and (3) oxidases that catalyze essentially irreversible oxidation of alcohols [1]. ADHs from category 1 have been divided into the following: group I long-chain (approximately 350 amino acid residues) zinc-dependent enzymes; group II short-chain (about 250 residues) zinc-independent enzymes; and group III “iron-activated” enzymes that generally contain approximately 385 amino acid residues [1,2]. ADHs’ functions are crucial to the fungal life cycle, such as growing under aerobic or anaerobic conditions, living inside the host, or establishing symbiotic interactions. With the increase in genome sequencing of fungal isolates, ADH systems in fungi belonging to different taxonomic groups have been revealed. However, progress in understanding the molecular structure and physiological role of the various ADH enzymes has been made in very few organisms.

This review focuses on the characteristics, regulation, and physiological role of fungal enzymes described in the literature, most of which belong to category 1 and a few to category 3 of oxidoreductases. Furthermore, we analyzed the protein entries annotated as “alcohol dehydrogenase” in the NCBI database. We observed that the freer the fungal lifestyle, the higher the amount of Zn-dependent ADH genes. The following sections review and update different aspects of zinc-dependent ADHs and zinc-independent enzymes described in the literature from yeasts, filamentous fungi, and other organisms. Aspects of interest include biochemical properties, the regulation of their production, role in energy metabolism, growth, morphogenesis, and pathogenesis, as well as the interaction between biological species, evolution, and biotechnological potential.

## 2. Physiological Role of ADH in Fungal Cells

The most well-studied ADHs for their role in growth and energy metabolism are ADHs from category 1, group I (Zn-dependent), and group II (Zn-independent proteins). Therefore, here, we summarize ADHs with known growth and energy roles.

### 2.1. Role of Zn-Dependent ADHs in Growth and Energy Metabolism in Yeasts

The yeast *Saccharomyces cerevisiae* is the organism in which the most progress has been made in relation to the identification and study of the function of ADH proteins so that the proteins encoded by the *ADH1* to *ADH7* genes have been identified and biochemically and physiologically characterized. *S. cerevisiae* is a Crabtree-positive organism in which the availability of oxygen is irrelevant to fermentative metabolism. Glucose generates high levels of glycolytic enzymes and represses respiration, which leads to ethanol production [3]. In this yeast, both in the presence and absence of oxygen, the cytosolic enzyme Adh1p produces ethanol and NAD^+^ using glucose as a carbon source. Under aerobic conditions, after glucose depletion, the ethanol produced is oxidized to acetaldehyde via the cytosolic enzyme Adh2p; this last compound can be metabolized via the tricarboxylic acid cycle or as an intermediate metabolite in gluconeogenesis [4,5]. In *S. cerevisiae*, the Adh3p enzyme is localized in the mitochondria and was proposed to be involved in the transport of mitochondrial-reducing equivalents to the cytosol [6]. By using quadruple mutants in *ADH* genes, in which only a single functional ADH was left, it was shown that the enzyme Adh3p produces ethanol from acetaldehyde with a capacity no greater than that shown by Adh2p. In the absence of glucose, Adh3p enables growth and ethanol assimilation, which indicates that the product of the *ADH3* gene replaces the Adh1p and Adh2p enzymes in its function, which occurs to a limited extent and is only noticeable when these enzymes are not active [5]. The Adh4p protein is not similar in amino acid sequence or structurally with the enzymes Adh1p, Adh2p, and Adh3p; it is activated by zinc and resembles Adh1p in its kinetic parameters [7]. Subsequent studies, using a quadruple mutant that only has Adh4p as a functional enzyme, showed that this enzyme does not allow the production of ethanol via the reduction of acetaldehyde during growth in glucose medium. A similar experimental approach showed that the enzyme Adh5p does not support growth in ethanol as the sole carbon source, indicating that this enzyme is not appreciably involved in the oxidation of ethanol to acetaldehyde [5]. The *ADH6* gene from *S. cerevisiae* encodes a zinc-containing ADH. The enzyme showed specificity to NADPH and activity with aldehydes and primary, aliphatic (linear and branched chain), and aromatic alcohols. The interruption of the *ADH6* gene makes *S. cerevisiae* sensitive to toxic concentrations of veratraldehyde, and the overexpression of the *ADH6* gene enables the yeast to grow under these conditions [8]. The *ADH7* gene of *S. cerevisiae* codes for an NADP(H)-dependent ADH, like the Adh6p enzyme. The purified Adh7p enzyme is a homodimer that exhibits broad substrate specificity similar to Adh6p. The deletion of the *ADH7* gene does not affect growth, although with the mutant deleted in this gene, no experiments were carried out on the phenotype of sensitivity to compounds such as veratraldehyde and anisaldehyde, which are produced during ligninolysis and substrates for the enzymes Adh6p and Adh7p [8].

In the galactose-fermenting yeast *Kluyveromyces lactis,* four *ADH* genes have been identified that encode cytosolic (*Kl*Adh1p and *Kl*Adh2p) and mitochondrial isozymes (*Kl*Adh3p and *Kl*Adh4p) [9,10,11]. The physiological role of *K. lactis* ADH enzymes was investigated by generating triple-mutant strains in *ADH* genes, in which only a single functional ADH was left [12]. The analysis of these mutants showed that each ADH activity enables growth in ethanol as the sole carbon source; only the strain carrying all four mutant genes showed an inability to grow in ethanol. Regarding the accumulation of ethanol produced in a culture medium containing glucose, different amounts were observed, *Kl*Adh1p being the highest producer, followed by *Kl*Adh2p, and finally, *Kl*Adh3p and *Kl*Adh4p produced ethanol at a very low level [12].

The yeast *Pichia stipitis* (currently known as *Scheffersomyces stipitis*) is one of the few organisms capable of fermenting the pentose xylose, although it is also capable of using glucose; the yeast shows the regulation of the transition between the respiratory and fermentative processes [13]. *S. stipitis* is a Crabtree-negative organism, so oxygen limitation, rather than the presence of glucose or xylose, induces fermentation [14,15]. Using gene-disruption experiments, the physiological role of the *ADH*-gene-encoded products was studied. The loss of *Ss*ADH1 negatively affects growth and ethanol production in a xylose medium under oxygen limitation [16]. On the other hand, the loss of *Ss*ADH2 enzyme function did not cause the aforementioned effects. The double-mutant strain *Ss*ADH1 *SsADH2* could not grow in xylose and produced very little ethanol. These observations suggested that the *Ss*ADH2 isoenzyme is also involved in xylose fermentation [16]. Like *S. stipitis*, *Candida maltosa* is capable of fermenting glucose or xylose. In this yeast, three ADH genes, *CmADH1, CmADH2A*, and *CmADH2B,* were cloned and sequenced [17]. In a mutant of *C. maltose* isolated as resistant to allyl alcohol, it was observed that the activity of the *Cm*ADH2A enzyme was affected and that this deficiency caused a dramatic decrease in ethanol production in a medium with xylose under oxygen limitation; however, the above-mentioned deficiency of *Cm*ADH2A enzyme activity had no effect on ethanol production from glucose under aerobic conditions [17]. According to the expression pattern of *Cm*ADH isozymes and fermentation studies of the *Cm*ADH2A-deficient mutant, it was found that *CmADH1* and *CmADH2A* were both expressed and involved in ethanol formation during xylose metabolism [17]. *Candida utilis* has also been known as a yeast capable of metabolizing xylose; the enzyme ADH1 has been described in this organism. Inspection of the amino acid sequence of *C. utilis* ADH1 and enzyme characterization with different aliphatic and branched alcohols indicated that it could be a primary alcohol dehydrogenase that requires zinc ions for catalytic reactions; the specific ADH activity is nine times higher for NAD^+^ than that for NADP^+^, indicating that the enzyme preferred NAD^+^ to NADP^+^ as a cofactor [18].

The yeast *Yarrowia lipolytica* can utilize ethanol as a carbon source but is not capable of producing ethanol or growing under anaerobic conditions. Eight *ADH* genes were identified in this yeast, all of which were deleted [19,20]. The characterization of combinations of mutations in the *ADH* genes showed that the joint alteration of the *ADH1*, *ADH2*, and *ADH3* genes results in the phenotype of an inability to grow in ethanol, indicating that the corresponding proteins participate in the oxidation of the alcohol and that it is enough that one of them is active for this process to occur. Additionally, it was observed that for growth in ethanol, in addition to the three *ADH* genes mentioned, the *ACS1* gene is required, which encodes an acetyl-CoA synthetase [21]. On the other hand, another study found that alcohol dehydrogenase genes *ADH1* and *ADH3* and a fatty alcohol oxidase gene, *FAO1*, play an important role in the oxidation of exogenous fatty alcohols but play less prominent roles in the oxidation of fatty alcohols derived from *n*-alkanes [20].

### 2.2. Role of Zn-Dependent ADHs in Growth and Energy Metabolism in Filamentous Fungi

*Neurospora crassa* was one of the first filamentous fungi in which the ability to produce appreciable amounts of ethanol was demonstrated in cultures incubated in a glucose medium [22]. Through zymography, it was shown that the fungus produces two different ADHs. Fermentative ADH, which showed a single band of protein with activity, is produced when the fungus is cultured in sucrose, while oxidative ADH, which consists of at least two electromorphs, is produced in a medium with ethanol as a carbon source; the latter enzyme is repressed by glucose or by sucrose. The enzymes also differ in substrate specificity, in the ratio of the forward (glucose as substrate) and reverse reactions (ethanol as substrate), and in their thermostability [23]. It has been shown that *N. crassa* can produce ethanol in cultures with different hexoses and pentoses, as well as with cellulose polymers and with lignocellulosic agroindustrial residues, such as straw, wood, and various agricultural and wood processing waste products [22]. Genes encoding *ADH1* and *ADH3* enzymes have been identified and expressed in *E. coli* to carry out the biochemical characterization of the enzymes [24]. However, no alteration experiments have been carried out on these genes to demonstrate in vivo the function of the enzymes they encode.

In *Aspergillus nidulans*, three genes that code for ADH enzymes have been identified. The *alcA* gene codes for the enzyme ADHI, which is the enzyme required to utilize ethanol as the sole carbon source [25,26,27]. The enzyme alcohol dehydrogenase II (ADHII) has been detected, by its activity, in zymograms, although its physiological function is unknown. This enzyme is encoded by the *alcB* gene [28]. The gene *alcC* encoding the enzyme alcohol dehydrogenase III (ADHIII) was identified as a cDNA sequence capable of complementing the *ADHI* mutation in *S. cerevisiae* [29]. To date, there is no known physiological function of the enzyme.

In *Aspergillus flavus* Furukawa et al., [30] reported that ethanol and 2-propanol at low concentrations (<1% and <0.6%, respectively) increase aflatoxin production. Both alcohols are incorporated into the aflatoxin biosynthesis via acetyl-CoA. In this context, *A. flavus* utilizes ethanol and 2-propanol as carbon sources for aflatoxin biosynthesis, and the *adh1* gene and probably other putative alcohol dehydrogenase controls this aflatoxin production, balancing ethanol production and catabolism.

*Mucor circinelloides f. lusitanicus* (currently known as *Mucor lusitanicus*) is a dimorphic fungus belonging to the subphylum Mucoromycotina. It has been described that *M. lusitanicus* is a Crabtree-positive organism since, when grown in a high-glucose medium under aerobic conditions, it produces appreciable amounts of ethanol [31,32]. In this organism, the *adh1* gene was identified, which encodes a zinc-dependent enzyme that uses NAD^+^ as a cofactor [33]. A mutant of *M. lusitanicus* altered in the *adh1* gene showed, in comparison with the wild-type strain R7B and with the mutant complemented with the wild-type *adh*^+^ allele, alteration to several physiological characteristics, including an inability to grow under anaerobic conditions in a glucose medium, production of low levels of ethanol in a medium with glucose in cultures carried out under aerobic conditions, a notable reduction in growth in a medium with ethanol as the only carbon source under aerobic conditions, and very low levels of alcohol dehydrogenase activity in the cytosolic fraction of the mycelium obtained under aerobic conditions in a glucose medium. These observations indicate that in *M. lusitanicus,* the product of the *adh1* gene acts to mediate the Crabtree effect and can act as a fermentative or oxidative enzyme, depending on the nutritional conditions [34].

Shah et al. [32] suggests that the *adh1* gene from *M. circinelloides* is also involved in lipid biosynthesis. In the *M. lusitanicus* strain WJ11, the knocking out of the *adh1* gene reduced the ethanol production by 85–90%, and the lipid and fatty acid content was decreased. These lipid and fatty acid contents were restored when the fermentation media was supplemented with 0.5% external ethanol.

In the phytopathogenic fungus *Fusarium oxysporum* f. sp. *lycopersici*, mutants deficient in the *ADH1* gene were isolated by obtaining spontaneous allyl alcohol-resistant mutants in a glucose medium. The characterization of one of these strains showed that the insertion into the *ADH1* gene of an incomplete transposable element of *F. oxysporum* occurred. This mutation caused an inability to use ethanol as a carbon source under aerobic conditions, impaired growth under hypoxic conditions with glucose as a carbon source, and decreased ethanol production in the glucose medium. Complementation with the wild-type *ADH1* allele restored all defects produced by the mutation, indicating that depending on the culture conditions, the *ADH1* gene product has fermentative and oxidative enzymatic functions [35].

*Botrytis cinerea* is a necrotrophic fungus that infects many types of plant species. A *Bcadh1* gene was identified in this organism, encoding a zinc-dependent enzyme. A mutant strain deleted in the referred gene was obtained and characterized [36]. It was observed that, compared to the wild-type strain, the Δ*bcadh1* mutant showed several phenotypic alterations, including a reduced growth capacity in low oxygen tension, greater tolerance to ethanol, and sensitivity to reactive oxygen species. The increased ethanol tolerance of the Δ*bcadh1* mutant indicated that the enzyme is involved in the fermentation of glucose to ethanol. However, the phenotypic characteristic that best explains this physiological role of the enzyme is the lower growth capacity in low oxygen tension [36].

Table 1 summarizes the substrates, cofactors, oligomeric state, cellular location, and function of fungal Zn-dependent ADH enzymes.

### 2.3. Role of Non-Zn-Dependent ADHs in Growth and Energy Metabolism

The white rot basidiomycete fungus *Phanerochaete chrysosporium* can degrade lignin to carbon dioxide, which requires enzymes that carry out the reduction of aryl-aldehydes to the corresponding alcohols. In this organism, the *PcAAD1* gene encoding a 386-amino-acid aryl alcohol dehydrogenase (AAD) was identified. The *PcAAD1* gene is expressed in a higher proportion under conditions of nitrogen limitation [43,45]. Analysis of the protein *Pc*Aad1p indicates that it belongs to the AKR9A subfamily of the AKR (aldo-keto reductase) superfamily, in which there is a conserved NADPH-binding domain. The purified enzyme is active in the reduction of more than 20 different aliphatic and aryl aldehydes, although it shows the highest activity with veratryl alcohol, vanillyl alcohol, and the corresponding aldehydes [43]. A similar AAD enzyme is overexpressed in the white rot basidiomycete *Coriolus versicolor* exposed to either 4-methyldibenzothiophene-5-oxide (4MDBTO) or dibenzothiophene-5-oxide (DBTO) [46]. It is proposed that in these organisms, aryl alcohol dehydrogenase is part of the lignin degradation machinery and the response to chemical stress [43,46].

Anaerobic chytridiomycete fungi, such as *Piromyces* and *Neocallimastix*, are important symbionts in the gastrointestinal tracts of herbivorous mammals, where they contribute to the degradation of plant polymers that form an important part of the diets of ruminants [47]. It was shown that *Piromyces* and *Neocallimastix* have an *ADHE*-type gene in their genome and that the ADHE protein was located in the cytosol [47]. This enzyme is homologous to the ADHE protein of eubacteria and that from parabasal flagellate anaerobes *Trichomonas vaginalis* and *Tritrichomonas fetus* [48]. *Piromyces* sp. E2 conducts the final steps of its carbohydrate catabolism via PFL and ADHE enzymes, producing metabolites of a bacterial-type mixed-acid fermentation. Similar to bacterial ADHE, the *Piromyces* ADHE enzyme is anticipated to be iron-dependent for catalysis and uses NAD as a cofactor [49].

## 3. Regulation of the Fungal ADHs

The transcriptional regulation of yeast and filamentous fungal ADHs involves different factors, including the carbon source, oxygen, morphogenesis, and host organisms’ infection processes. Some examples of this regulation are discussed below.

### 3.1. Regulation by Carbon Source

ADH regulation by the carbon source has been broadly studied in yeast and filamentous fungi and is the primary transcriptional regulation mechanism. The *S. cerevisiae* genome contains 16 genes that encode ADHs that catalyze the interconversion of aldehydes and alcohols [50]; five of them (*ADH1* to *ADH5*) are involved in ethanol metabolism [51]. Adh1p is the major ADH activity during anaerobic fermentation [52]. The *ADH1* gene is highly expressed in cells grown in media with glucose as a carbon source and repressed when the cells are grown in a medium with a nonfermentative carbon source such as ethanol [53]. *ADH1* up-regulation requires activation by the transcription factors Gcr1p (glycolysis regulator) and Rap1p (repressor/activator protein), both probably forming a complex in vivo [54,55]. The *ADH1* gene is also repressed via zinc starvation [56].

The regulation of the *ADH2* gene is one of the most studied in yeast; the gene is regulated by catabolite repression; when glucose is depleted in the medium, the Zn-finger DNA-binding protein Adr1p and the DNA-binding protein Cat8p bind in the specific regions of the *ADH2* promoter [57,58]. Adr1p binds to a 22 bp sequence present in the upstream activation sequence (UAS1) [57], and Cat8p to the CSRE (carbon source-responsive element) [58]. The global response to glucose depletion is mainly initiated through a signal-transduction cascade by the Snf1p kinase complex, which phosphorylates serine and threonine residues; Adr1p and Cat8p are two of the transcription factors regulated by Snf1p [58]. Snf1p positively regulates Adr1p binding in the absence of glucose, while the type 1 protein phosphatase (PP1) complex Glc7p/Reg1p inhibits Adr1p binding in the presence of glucose [59]. There are other elements involved in *ADH2* gene regulation. Chromatin modification is required and affected by Adr1p binding, the loss of histone deacetylase activity allows Adr1p to bind promoters under repressing conditions, and Adr1p has been shown to mediate nucleosome repositioning in the *ADH2* promoter [60]. Additionally, de Smidt et al. [4] listed at least 24 genes that influence the *ADH2* gene expression, and Simpson-Lavy and Kupiec [61] reported that *ADH2* expression is also inhibited by acetate.

Adh3p is a mitochondrial ADH [62]. The *ADH3* gene, similar to the *ADH2* gene, is repressed by glucose [62,63]. The regulation of the *ADH4* and *ADH5* genes has not been exhaustively studied. The *ADH4* gene is induced in response to zinc deficiency [64]. The *ADH4* gene is up-regulated by lithium in yeast cells grown in galactose. Lithium becomes toxic to yeast when grown in a medium containing galactose as a carbon source, inhibiting the phosphoglucomutase enzyme involved in galactose metabolism [65]. In most laboratory strains, the *ADH4* gene is expressed at low levels; however, Mizuno et al. [66] reported high expression of the *ADH4* gene during fermentation, in a brewer mutant strain resistant to 2-deoxyglucose, with high ethanol productivity. The 2-deoxyglucose is a nonmetabolizable glucose analog widely used to isolate mutants that lack glucose repression in *S. cerevisiae* [66]. The *ADH5* gene is a paralog of the *ADH1* gene, but contrary to *ADH1*, it is not regulated by the carbon source. Zhan et al. [67] analyzed the global gene expression profile of *S. cerevisiae* through microarrays in response to high concentrations of the organic solvent dimethyl sulfoxide (DMSO). The studies showed that DMSO significantly regulates 1338 genes. In these analyses, it is observed that the *ADH4* gene is down-regulated; on the contrary, the *ADH5* gene is up-regulated. In this sense, the *ADH5* gene could be implicated in the cellular response against the toxic effect of DMSO.

In the yeast *K. lactis*, four *ADH* genes have been identified and characterized [9,10,68]. Carbon sources transcriptionally regulate the four genes. The *KlADH1* and *KlADH2* genes are more expressed in a medium containing glucose with respect to cells grown in ethanol [69]. The *KlADH3* gene is repressed by ethanol, while *KlADH4* is induced by ethanol added to the medium or produced during fermentation [70,71].

Bertram et al. [72] reported that in *Candida albicans*, *ADH1* gene expression is regulated in response to a carbon source. However, Bakri et al. [73] reported that the *ADH1* gene is constitutively expressed in different media. In this study, *ADH2* gene expression changes with the growth phase and culture medium, being more expressed at the stationary phase in cells grown in a rich medium, with no detectable expression of the *ADH3* gene [73]. The expression of the three *ADH* genes of the xylose-fermenting yeast *C. maltose* (*CmADH1*, *CmADH2A*, and *CmADH2B*) is like the *Saccharomyces* ADH system, where *CmADH1* is induced in the presence of glucose. Meanwhile, *CmADH2A* is expressed after the glucose is consumed, and *CmADH2B* shows low expression [17].

In *A. nidulans*, three NAD^+^-ADHs can utilize ethanol as a substrate. The AdhIp encoded by the *alcA* gene is the physiological enzyme of ethanol catabolism [27]]; AdhIIp is encoded by the *alcB* gene [74], and AdhIIIp is encoded by the *alcC* gene [39]. The *alcA* gene is induced by ethanol and regulated by repression catabolism [75], and the *alcB* gene is induced by carbon starvation [38]. In general, in *Aspergillus,* the *alc* system is directed by two regulatory circuits controlling ethanol catabolism, the transcriptional activator AlcR and the repressor CreA [76,77]. AlcRp is necessary for the transcription of *alcA* and stimulates its own expression [78]. The effector of AlcR is acetaldehyde [79]. CreA mediates carbon catabolite repression, directly repressing AlcR and alcA [79].

### 3.2. Regulation by Oxygen

The fungal *ADH* genes are not exclusively regulated by the carbon source; oxygen is also involved in the transcriptional regulation mechanism. In the xylose-fermenting yeast *S. stipites,* the ADH1 and ADH2 amino acid sequences have a high similarity to those of *Sc*Adh1p [16], but the regulation of the *SsADH1* and *SsADH2* genes is distinct. Contrary to the glucose-dependent regulation of the *ScADH1* and *ScADH2*, the *SsADH1* gene is regulated transcriptionally by oxygen; in cells grown in xylose as a carbon source and under oxygen-limited conditions, *SsADH1* expression is higher than in aerobic conditions, and the expression correlates with the oxygen concentration, where the expression increases as oxygen decreases [80]. Meanwhile, *PsADH2* is not expressed under these conditions [80]. The expression of the gene *SsADH2* is elevated in mutant cells with the disruption of the *SsADH1* gene, grown on ethanol or glycerol but not in xylose under oxygen-limited conditions [80].

Other examples of ADH gene regulation by oxygen are found in filamentous fungi. Corrales Escobosa et al. [35] reported that the *F. oxysporum* f. sp. *lycopersici Adh1* gene is up-regulated in mycelium grown under hypoxic conditions. In *A. nidulans*, the *alcC* gene encoding AdhIIIp is regulated at both the transcriptional and translational levels, induced by anaerobic stress, and required for long-term survival under these anaerobic conditions [39]. In the entomopathogenic fungus *Metarhizium acridum,* the *Adh1* gene is similarly regulated by oxygen; under hypoxic conditions, the *Adh1* gene is up-regulated [40].

### 3.3. Regulation by Morphogenesis

Members of the genera *Mucor* have yeast–hyphae dimorphism; in some of them, such as *M. lusitanicus* and *M. rouxii*, this morphogenetic process is influenced by carbon metabolism [33,81,82]. In *Mucor*, the role of ADH during yeast–hyphae dimorphism has been studied. In *Mucor*, anaerobic yeast is associated with high rates of ethanol production [83], which correlate with higher levels of ADH activity [84]. *M. rouxii* shows elevated ADH activity in mycelium cells grown in the absence of oxygen, whereas in yeast cells, ADH activity is not dependent on the presence or absence of oxygen [85]. Under aerobic conditions, in the presence of glucose, *M. lusitanicus* grows as mycelium and produces significant amounts of ethanol [33]. There is no difference in *Adh1* gene expression between aerobic mycelium and anaerobic yeast cells when glucose is present. However, under aerobic conditions, the level of *Adh1* transcript correlated with the glucose concentration [33]. In this sense, Rangel-Porras et al. [34] suggested that the regulation of the *adh1* gene is not influenced by morphology or oxygen concentration but instead depends on nutritional conditions.

### 3.4. ADH Gene Regulation during the Infection Process

In the entomopathogenic fungus *Metarhizium anisopliae,* the *Adh1* gene is expressed during the infection of the insect *Plutella xylostella* [41]; this expression could be the result of hypoxic conditions inside the insect. The authors suggested that Adh1p activity is required to penetrate and colonize *P. xylostella* larvae. However, Zhang et al. [40] reported that in *M. acridum,* the *Adh1* gene is not involved in the virulence of *Locusta migratoria*. In *C. albicans,* [86] reported reduced *ADH1* gene expression and a lower presence of Adh1p in *Candida* biofilm cells than in planktonic cells. In the same way, a strain with a disruption of the *ADH1* gene forms thicker biofilms in vitro and in vivo, with an increased capacity to invade and damage host tissues [86]. In murine models of invasive pulmonary aspergillosis, *Aspergillus fumigatus* is exposed to hypoxia; under these conditions, the *alcC* gene shows increased expression in vitro and in vivo during fungal pathogenesis [87]. In vivo, the primary role of the *M. lusitanicus* ADH1 enzyme is to convert acetaldehyde to ethanol [33]. The *M. lusitanicus Adh1* gene is not only involved in fungal morphology and metabolism. A mutant in the *Adh1* gene with reduced ADH activity accumulated higher acetaldehyde concentrations compared with the parental wild type and showed higher virulence in a mouse infection model. These effects can be attributed to acetaldehyde overproduction [81].

### 3.5. Novel ADH Gene Regulation

In *Candida utilis,* Tomita et al. [88] performed a genome and transcriptome analysis using next-generation sequencing and found an antisense transcript of the *ADH1* gene, transcribed in the stationary phase. The antisense transcript *Cu*ADH1-R is expressed during the stationary phase but not the log phase, contrary to the sense transcript *Cu*ADH1-R. The inverse correlation between the expression of these two overlapping transcripts suggests a possible mechanism whereby the expression of the antisense transcript acts to repress the expression of CuADH1-F, repressing the ethanol production during the stationary phase [88]. Although this *ADH* antisense transcript has not been described in other fungal systems, it would be interesting to analyze fungal transcriptomes and determine whether it exists. This mechanism could regulate *ADH* gene expression and create a novel regulation system for ethanol production.

The regulation of fungal ADH is complex; the examples shown are just some of the several factors that regulate the expression of *ADH* genes, their importance, and the role that these genes play in the life cycles of yeasts and filamentous fungi, as well as their influence on pathogenicity and virulence. Continuing the study paves the way for new areas of application and understanding of the marvelous world of yeast and filamentous fungi. Table 2 summarizes the factors involved in *ADH* gene regulation.

## 4. Role of ADH in Pathogenesis and Morphogenesis

Fungal growth during the infection process needs to adapt to variable oxygen levels, and these levels drop from 21% in the atmosphere to 2% or even less in healthy human tissues; moreover, the infection process leads to a decrease in oxygen concentrations [89]. In this scenario, the control of oxidative–fermentative metabolism is critical for fungal growth during the infection event. For example, in dimorphic species of *Mucor*, growth under anaerobiosis stimulates the fermentative metabolism; meanwhile, the presence of oxygen favors the oxidative metabolism [83]. Fungal pathogens need to regulate the rate of oxidative–fermentative metabolism to adapt to changes in oxygen levels. In this context, ADHs play a pivotal role, allowing the regeneration of NAD^+^ to continue along the glycolytic pathway for energy production under low oxygen levels or a lack of oxygen [90,91].

### 4.1. Role of ADH in Human Fungal Pathogens

Although the infection process requires a successful host–tissue invasion by the fungal pathogen, in some cases, there is a lack of understanding of the role of ADH in the virulence, as has been reported in *A. fumigatus*, in which the function of the fermentative ADH has been described as not being involved in virulence. In this ascomycete, from the three-alcohol dehydrogenase-encoding genes reported (*alcA*, *alcB*, and *alcC*), only the mRNA of *alcC* is highly accumulated under hypoxic conditions, and its product is needed for ethanol production. The deletion of *alcC* did not affect the survival of the chemotherapeutic and X-linked chronic granulomatous disease mouse models compared to those animals infected with the wild-type strain. Mice infected with Δ*alcC* and previously treated with the corticosteroid triamcinolone to cause a hyperinflammatory response exhibit mortality rates comparable to those of the wild-type strain. Compared to the wild-type strain, the fungal burden in the lung tissue from mice treated with triamcinolone was lower, but an intensified inflammation response was observed for those animals infected with Δ*alcC*, indicating that *alcC* is not critical for the death of the animals but increases lung inflammation. However, it is still probable that one of the other *alc* genes could replace the function of *alcC* [87].

In the dimorphic ascomycete *C. albicans*, deleting the *ADH1* gene decreased alcohol production and increased biofilm formation via acetaldehyde accumulation [86]. The deletion of the *ADH1* gene decreased the virulence of *C. albicans* in mice, in the nematode, and in *Galleria mellonella* [92]. In the same context, the deletion of *ADH1* in *C. albicans* inhibits hyphal development and mitochondrial oxidative phosphorylation; it is probable that lower energy production results in a delay in vegetative development [90]. The pivotal role of ADH in the control of the fermentative metabolism could constitute a critical step for the success of the infection process. Moreover, it has been described that host plasminogen can be bound to the cell wall proteins of *C. albicans* that might contribute to host–tissue invasion; by proteomic analysis, alcohol dehydrogenase was found to be a major plasminogen-binding protein isolated from the cell wall. The protease plasmin is generated following the binding of plasminogen to the cell wall, most likely by a host activator. Although plasmin is able to degrade fibrine and may contribute to increasing virulence, endothelial cell injury was not noticeable [89]. In general, controlling the activity of this enzyme might be a potential target for inhibiting the fungal infection event.

In the dimorphic *M. lusitanicus* [93], during tissue invasion, the fungus needs to adapt to low oxygen availability, increasing the fermentative metabolism, but the *adh1* mutant of *M. lusitanicus* can enhance tissue invasion [81]; despite this, this mutant is not able to grow during the anaerobic-growth phase [34]. It is possible that the *adh1* mutant strain of *M. lusitanicus* continues to sense certain components from the host that enhance mycelial growth even under low oxygen conditions because it has been reported that *M. lusitanicus,* in the presence of blood serum, stimulates hyphal growth and virulence [94]. Moreover, the *adh1* mutant of *M. lusitanicus* cannot develop yeast cells under anaerobic conditions due to the role of this enzyme in the reoxidation of NAD^+^ [34]. Based on this information, it is possible that, depending on the intrinsic fermentative–oxidative metabolism, the ADH activities of the fungal pathogens could undergo different scenarios and host components could participate in regulation, as well as in the fungal metabolism.

### 4.2. Role of ADH in Fungal Plant Pathogens

In *B. cinerea,* the *bcadh1* gene is up-regulated during the tomato–*B. cinerea* interaction and at the early stage of infection. The mutant Δ*bcadh1* showed a smaller size of conidia with morphological change, less conidia formation, lower germination rate, and branching, and it was more sensitive to H_2_O_2_, SDS, and hypoxia conditions than the wild-type (WT) strain and complemented strain. On the other hand, during tomato leaf infection, the mutant Δ*bcadh1* caused a minor lesion compared to the WT and complemented strains. In summary, the ADH activity in *B. cinerea* is needed for development, adaptation, and virulence [36]. The deletion of *adh1* from the phytopathogen *F. oxysporum* f. sp. *lycopersici* caused less of a harmful effect during tomato plant infection [35]. In these two fungi, the mutation of *adh* led to a lower virulence rate, probably due to the lessened growth under hypoxic conditions during infection. In contrast, the silencing of an NADPH–cinnamyl alcohol dehydrogenase *Ss*CAD from *Sclerotinia sclerotiorum* presented similar virulence and hyphal growth compared to a WT strain. However, the silencing mutants in *Ss*CAD cause developmental delay, reduced formation of sclerotia and the survival structure of the fungus, as well as the down-regulation of *nox1*, *nox2*, and *noxR*, which encode ROS-generating NADPH oxidase. The phenotypes of *nox1*-, *nox2*-, and *noxR*-silencing mutants are comparable to those of the *Ss*CAD-silencing mutant. Normal sclerotia formation was restored by the addition of exogenous oxidants (H_2_O_2_) or NADPH, but not NADP^+^ or NADH, indicating that *Ss*CAD is linked to NADP^+^ oxidase and contributes to the maintenance of adequate intracellular levels of NADPH [95].

### 4.3. Roles of Fungal ADH in the Infection Process

As mentioned previously, the *Adh1* transcript in the entomopathogen *M. anisopliae* is highly accumulated during the insect-infection process and is needed for the full virulence phenotype [41]. In general, in some pathogens, ADH activity is needed for an adequate virulence phenotype, but this enzyme is needed for adequate adaptation, growth, and differentiation events. The latter has been described for *Adh1* mutation in *M. acridum*, which correlated with a lower growth rate and spore production only under hypoxic conditions, but no defects were observed under normal conditions [40]. Table 3 summarizes the roles of ADHs in biological interactions.

## 5. Role of ADH in Complex Biological Interactions

Alcohol and its metabolism play essential roles in the establishment and maintenance of biological associations. We have previously described its relevance to pathogenic activities. However, they are not the only interactions where the participation of ADH enzymes has been documented. Filamentous fungi, such as the entomopathogen *Metarhizium,* must deal with particular conditions of nutrients and oxygen availability when penetrating their insect hosts. There are no exact data related to these in vivo parameters. However, the activity and presence of ADH enzymes have been described in both aerobic and microaerobic in vitro conditions. As previously mentioned, in *Metarhizium,* the ADH enzyme is necessary for pathogenesis [41]. For *M. anisopliae* and for other *Metarhizium* species, it has documented activity as a plant endophyte (*Arabidopsis,* maize, and tomato), conferring improvements in growth, vigor, and plant defense [100]. It is probable that ADH activity could also participate during plant tissue colonization, where intra- or intercellular micro-environments such as those previously described could be generated.

Biological interactions are much more complex and involve more participants. For instance, one of the most obvious examples of the relevance of ADH activity to symbiont selection is demonstrated in ambrosial beetles. These insects farm their own fungi, which they feed on and depend on to develop and reproduce properly. It is an obligate mutualistic relationship, with fungi belonging to the genera *Ambrosiella* and *Raffaelea*. The beetles carry the fungi in structures known as mycangia; adult insects inoculate fungi while excavating complex galleries on host tree tissues, which contain ethanol. In this process, they can also inoculate other fungi (*Aspergillus*, *Paecilomyces*, and *Penicillium*) and bacteria, which are considered contaminants that compete with the mutualists, threatening the viability of ambrosial gardens. A way to study the success of mutualist gardens inside the galleries is to analyze the effect of ethanol on the growth of both kinds of fungi, mutualistic and contaminants. *Ambrosiella* and *Raffaelea* species can grow, while the competitors *Aspergillus* and *Penicillium* do not grow. Additionally, there is a correlation between this growth and the detected ADH activity. These data indicate that there is an advantage for ambrosial beetles in selecting an ethanol-rich substrate to grow their fungal symbionts, as ethanol functions as an antiseptic against unwanted competing fungi and allows for the growth of the mutualistic symbionts, which possess the necessary metabolism to survive. This work highlights an example of competition-based selection where hosts can maintain mutualistic associations with beneficial symbionts by creating a demanding environment such that the host-preferred symbiont can better withstand the demands [99].

This competitive capacity of mutualistic symbionts is also present in other species of beetles, including the genus *Dendroctonus* [101], and suggests that there could be more cases of selection based on competition between biological associations by taking advantage of ADH activity and participation of the coevolution of symbionts. *Dendroctonus* spp. bark beetles participate in the forest regeneration cycle by colonizing dead or weakened pine trees. However, sometimes, there may be uncontrolled outbreaks that alter the landscape and ecological balance. For this reason, it is important to study this association and its key elements. The colonization of trees by beetles requires that they be able to confront the plant’s chemical defense, which consists of a mixture of terpenes, among various other compounds. Part of the strategy of *Dendroctonus* spp. to deal with toxic compounds is the establishment of complex associations with several microorganisms, whose metabolic functions are used by the beetles. The yeast *Cyberlindnera americana* is the predominant microorganism in the gut of *Dendroctonus* spp., and the transcriptomic analysis of the yeast exposed to α pinene, the main terpene present in pine trees, shows an increase in the expression of genes related to the detoxification process, among which those that encode for aryl-alcohol dehydrogenases are notable. In this tripartite interaction, the evidence suggests that the activities carried out by the products of these genes could enable yeasts to resist high amounts of terpenes inside beetles during the colonization of pine trees [98].

## 6. Zinc-Dependent ADHs in Fungal Genomes

As of June 2023, in the NCBI Reference Sequence (RefSeq) database, there are 933,766 protein entries annotated as “alcohol dehydrogenase” distributed in the tree of life. For fungi, there are 14 704 entries in the NCBI RefSeq database (NCBI Protein database, 2023). Fungal protein annotations have increased with genome sequencing. With the 1000 Fungal Genomes Project, MycoCosm was created as a fungal genomics portal (http://jgi.doe.gov/fungi (accessed on 22 June 2022)), developed by the US Department of Energy Joint Genome Institute [102]. Using the fungal tree generated in MycoCosm, we analyzed the distribution of zinc-dependent alcohol dehydrogenases encompassing the taxonomic classes of the fungal kingdom that have reference genomes (Table 4).

Thirty-seven species of fungi were analyzed, and all had at least one zinc alcohol dehydrogenase (Zn-ADH). The species with the most Zn-ADHs are *F. oxysporum* f. sp. *lycopersici* and *Basidiobolus meristosporus*, with 30 and 25, respectively. Species with one Zn-ADH include *Lactarius pseudohatsudake*, *Piptocephalis cylindrospora*, *Catenaria anguillulae*, *Piromyces finnis*, *Mitosporidium daphniae*, *Rozella allomycis*, and *Schizosaccharomyces pombe*. The analysis of Table 4 shows that there is no relationship between the size of the genome and the number of Zn-ADHs. As an example, we see that for *F. oxysporum* f. sp. *lycopersici*, the genome is 53.91 Mb with thirty Zn-ADHs. For *Gigaspora margarita*, the genome is 773.1 Mb with six Zn-ADHs.

We observed that the number of Zn-ADHs in fungal genomes is related more to their lifestyle than their genome size, i.e., the species with restricted lifestyles have a lower number of Zn-ADHs. One to four Zn-ADHs were found in *R. allomycis*, *Caulochytrium protostelioides*, *Dimargaris cristalligena*, and *P. cylindrospora* species, which are obligate mycoparasites [103,104]. Similarly, for *Mitosporidium daphniae,* only one Zn-ADH was observed, which is an obligate endoparasite of the epithelium of the lower midgut of the water flea *Daphnia magna* [105]. One and three Zn-ADHs are annotated for the fungi *P. finnis* and *Neocallimastix* sp., which are obligate anaerobes that inhabit the rumen of herbivorous animals [106,107]. The ectomycorrhizal fungi *Tuber indicum* [108], *L. pseudohatsudake* [109], and *Laccaria bicolor* [110] show one to two Zn-ADHs. The arbuscular mycorrhizal fungi (AMF) *G. margarita* has six Zn-ADHs [111].

We observed that in fungi with freer lifestyle capabilities, including saprophytes, the number of Zn-ADHs increases in their genomes similarly if the ability to be pathogenic is added. Among the most studied yeast saprophytes with most of their Zn-ADHs characterized, we have *S. cerevisiae* [112] and *K. lactis* [113], with 7 and 12 Zn-ADHs, respectively. *S. cerevisiae* is known to be found in plants in nature [114], and *K. lactis* has been isolated from soils [115]. In filamentous saprophytes such as *M. lusitanicus* [116], *N. crassa* [117], *Trichoderma reesei* [118], *A. nidulans* [119], and *B. meristosporus,* 6 to 25 Zn-ADHs are found in their genomes [120].

In fungi with the capacity to be saprophytic and pathogenic, a greater number of Zn-ADHs is also observed than in fungi with restricted lifestyles. However, the analysis of the number of ADHs in the fungal genomes shows that there seems to be no relation between the number of ADH genes and the lifestyles of the fungi. Nevertheless, this must be confirmed through experimentation since only in *S. cerevisiae* all Zinc-dependent ADHs have been studied. For the phytopathogenic fungi *Puccinia graminis* [121], *Ustilago maydis* [122], *Armillaria ostoyae* [123], *B. cinerea* [124], *Alternaria rosae* [125], and *F. oxysporum* f. sp. *lycopersici* [126], we observed 2 to 30 Zn-ADHs annotations in their genomes. A higher number of Zn-ADHs is also observed in the pathogenic fungi of animals. For the opportunistic pathogens *C. albicans* [127] and *Cryptococcus neoformans* [128], four and six Zn-ADHs were found, respectively. The entomopathogenic fungus *M. robertsii* [129] has eight Zn-ADHs. The fungal *Dactylellina haptotyla* pathogen of nematodes has four Zn-ADHs [130].

### Phylogenetic Tree of Fungal Zn-Dependent ADH

The zinc-dependent alcohol dehydrogenases are in the superfamily of MDR (medium-chain dehydrogenase/reductase). In 1983, ADH and YADH (yeast ADH) were the only MDR families. In 2007, the superfamily of MDR was made up of 17 families, including TDH (threonine DH), QOR (quinone oxidoreductase), VAT (vesicle amine transport), LTD (leukotriene DHs), ACR (acyl-CoA reductase), CAD (cinnamyl ADH), TADH (tetrameric ADH), and ADH [131]. The YADH family was renamed TADH (tetrameric ADH) due to its tetrameric nature and more members of different organisms that share characteristics [131]. In 2008, Persson et al. [131] reported rapid growth of family members through large-scale genome projects, with the MDR families having the most members being ADH, CAD, LTD, and TADH [131].

Currently, we can observe in the NCBI conserved domain database that the MDR superfamily has continued to evolve in such a way that it currently consists of 84 families and the TADH (tetrameric ADH) family has been discontinued (NCBI-CDD, 2023). The last update of the conserved domain database (CDD) was made in November 2020 [132]. Currently, Zn-ADHs continue in the MDR superfamily, and fungal Zn-ADHs are in the CAD (cinnamyl alcohol dehydrogenase) family, which is divided into four subfamilies CAD1, CAD2, CAD3, and CAD_like (NCBI-CDD, 2023). Initially, the CAD family was made up of proteins belonging to plants. The CAD members from plants are important in lignin biosynthesis in plant cell walls, and they reduce cinnamaldehydes into cinnamyl alcohols in the last step of the monolignol pathway [131]. This activity of reducing cinnamaldehydes has also been described for *ADH6* and *ADH7* from *S. cerevisiae* [8,133].

**Figure 1:**
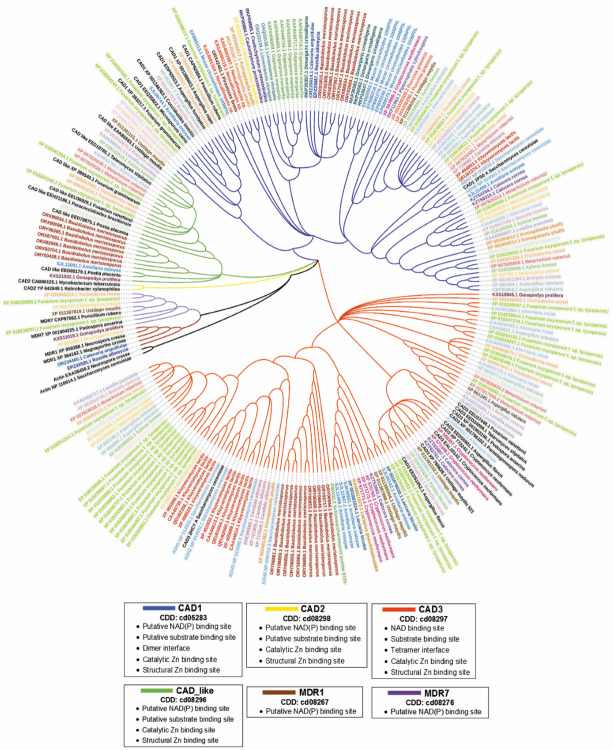
Phylogenetic tree of fungal Zn-ADHs. The phylogenetic tree is based on sequences of Zn-ADHs annotated in the NCBI databases. The Zn-ADHs annotated in the genomes of the 33 species shown in Table 4 were used. Each color in the names of the Zn-ADHs proteins corresponds to the same species. The names of the proteins in black correspond to reference Zn-ADHs of the MDR superfamily obtained from the NCBI Conserved Protein Domain Family database. The colors of the branches show belonging to the same family of domains. The four families of cinnamyl alcohol dehydrogenases (CAD) are shown: CAD1, CAD2, CAD3 and CAD_like. As an internal control of the MDR superfamily (medium-chain dehydrogenase/reductase), members of the MDR1 and MDR7 families were used. The branch in black corresponds to actin proteins used as an external group. The tree was inferred using the UPGMA method. The bootstrap consensus tree inferred from 10,000 replicates. This analysis involved 259 amino acid sequences. Analyses were conducted in MEGA11 [134]. For phylogenetic tree display and annotation, iTOLv6 was used [135].

To understand Zn-ADHs’ distribution in the fungal kingdom, a phylogenetic tree was created using Zn-ADHs from different representative species of the different taxonomic groups belonging to fungi (Table 4). The analysis of the phylogenetic tree of fungal Zn-ADHs (Figure 1) shows that Zn-ADH is distributed in three of the CAD subfamilies (CAD1, CAD3, and CAD_like), except for the CAD2 subfamily. The CAD subfamilies with the most members are CAD3, CAD1, and CAD_like, respectively. *Saccharomycetes* fungi only contribute Zn-ADH in the CAD3 and CAD1 subfamilies, in which tetrameric and dimeric Zn-ADH are found. The species that contribute members to the CAD1, CAD3 and CAD-like subfamilies are *U. maydis*, *B. cinerea*, *Lasallia pustulata*, *Xylona heveae*, *Alternaria rosae*, *M. robertsii, T. reesei, F. oxysporum* f. sp. *lycopersici, D. haptotyla*, *B. meristosporus*, and *A. ostoyae*. Most of the fungal species that have Zn-ADH members in the three CAD families interact with plants or vegetal material.

The phylogenetic tree of fungal Zn-ADHs generates more questions than answers, such as the following: Why do some species of fungi have so many Zn-ADHs in their genomes and others only one? Are all Zn-ADHs in the different genomes functional? What is the role of Zn-ADH in the various lifestyles of fungi?

As mentioned previously, *S. cerevisiae* has seven Zn-ADH [136], where *ADH6* and *ADH7* have enzymatic activity with cinnamyl alcohols, which are the immediate precursors of lignin. They also interact with lignin degradation products such as veratraldehyde, anisaldehyde, and vanillin [8,133]. The ability of *ADH6* and *ADH7* of *S. cerevisiae* to degrade lignin degradation products helps us to strengthen our proposal that there is a relationship between the number of Zn-ADHs present in their genomes and the different lifestyles of fungi. Zn-ADH may participate in the degradation of lignin in the case of fungal species that are known to have the capacity to do so. Similarly, Zn-ADH could help fungi establish themselves in ligninolytic environments, as proposed by Larroy et al. [8], since products derived from lignin biodegradation, such as vanillin, are toxic [137].

## 7. Biotechnological Applications of Fungal ADHs

People have used microorganisms and their products since ancient times, e.g., fermented beverages, which have played a significant role in human societies’ religious and cultural activities. *S. cerevisiae* is one of the best-known microorganisms in ethanolic fermentation and has been improved through metabolic engineering and adaptive evolution in the laboratory [138]. This example frames the importance of fungal ADHs for our society’s development.

### 7.1. ADHs in Biofuel Production

The study of fungal ADHs and their applications has a transcendent role to play in one of the critical issues in today’s world—renewable energy production, where ethanol production is overriding—without losing sight of the fact that these methodologies must have efficient productivity and be kind to the environment. Bioethanol, a renewable fuel, reduces vehicle emissions and improves air quality, a critical contribution to the ambient atmosphere and economy. Second-generation biofuels are relevant because they are produced using lignocellulosic biomass from agricultural and forestry waste. Microorganisms play a crucial role in the production of these biofuels.

Some of the main challenges are the hydrolysis of lignocellulose to release monomers of hexoses and pentoses (principal compounds in these residues) and avoiding the effect of toxic compounds that inhibit the growth and fermentation process of microorganisms. In the case of products of industrial interest generated by in vitro enzymatic cascade reactions, some problems are enzyme stability, reaction efficiency, and production yield. Many efforts have been made to solve these problems, and fungal ADHs are essential.

One strategy is the use of microorganisms adapted to grow in extreme conditions. In this sense, the unconventional yeast *Hansenula polymorpha* (*Ogataea polymorpha*), which belongs to a small group of methylotrophic yeasts, has excellent potential. It is a ubiquitous yeast found naturally in spoiled orange juice, cornmeal, some insects’ guts, and soil [139]. This organism can grow in methanol as the sole carbon source and tolerate and grow at high temperatures, over 50 °C [140]. In this yeast, the strain that overexpresses the mitochondrial *OpADH3* gene controlled by the constitutive p*GAP* promoter doubles ethanol production compared to the wild-type strain in a glucose medium. The highest ethanol yield of little more than 40 g.L^−1^ was observed on day 4. On the contrary, in a xylose medium, this strain reduced ethanol production six hundred times that observed in glucose. Moreover, the strain that overexpressed the *OpADH3* gene in this xylose medium produced six times less ethanol than the wild-type strain. These observations suggest that the modulation of OpADH3 gene expression would be a good objective for developing a better bioethanol producer from *O. polymorpha* on a specific substrate [141].

Another possibility is using supermicroorganisms to perform the complete processes, from the hydrolysis of lignocelluloses to bioethanol production. *N. crassa*, a saprophytic mycelial fungus and a natural inhabitant of the soil in tropical and subtropical regions [142], displays characteristics that indicate it is a good alternative in biofuel production [22]. *Neurospora* contains and expresses the complete enzymatic machinery to degrade raw materials and metabolize sugars rapidly via glycolysis and ethanolic fermentation, obtaining the desirable final product, biofuel ethanol, albeit with the presence of inhibitors of growth and fermentation [22]. The alcohol dehydrogenase coded by the *ADH3* gene is involved in ethanol production and is regulated by glucose. This *Nc*ADH3 has >50% identity with the *S. cerevisiae* ADH1, ADH2, and ADH3 enzymes [22] and has the same fermentative function as *Sc*ADH1p. However, *Neurospora* has lower fermentation efficiency than *Saccharomyces*. So, this *Nc*Adh3 gene can be a potential target to improve the efficiency and yield of bioethanol production in *Neurospora*.

The pretreatment of lignocellulosic biomass produces undesirable inhibitors like furfural and 5-hydroxymethylfurfural (HMF). The yeast *S. stipitis*, a natural inhabitant of the gut of *Passalidae* beetles, is very well known for its capacity for pentose fermentation and shows the best yield in the ethanolic fermentation of biomass [143]. Because of these characteristics, *S. stipitis* is relevant in the biofuel and bioenergy industries. Ma et al. [144] described that this yeast expresses multiple *ADH* genes involved in detoxifying aldehyde inhibitors derived from lignocellulosic conversion. *SsADH4* and *SsADH5* exhibited significant induction in the presence of aldehyde inhibitors, displaying a reduction of furfural and HFM to less toxic compounds FM (furan methanol) and FDM (furan-2,5-dimethanol), and showed the highest catalytic efficiency. Some of the other ADHs reduced other aldehydes (isovaleraldehyde, benzaldehyde, and phenylacetaldehyde), indicating that these ADHs have a broad range of aromatic and aliphatic aldehydes as substrates. So, *S. stipitis* shows promise in its ability to improve the fermentation of lignocellulosic biomass because the ADHs of this yeast eliminate aldehyde inhibitors, diminishing the stressful conditions for the microorganisms using these residues to grow and produce biofuels.

### 7.2. Role of ADH in Pharmaceutical and Cosmetic Industries

Fatty alcohols are relevant, especially in the pharmaceutical and cosmetic industries. Zhou et al. [145] described that in *S. cerevisiae*, two *ADH* genes, *ADH5* and *ADH6*, are relevant to fatty alcohol synthesis such as in 1-tetradecanol, 1-hexadecanol, and 1-octadecanol, among others. Using metabolic engineering, including the simultaneous overexpression of the *ADH5* gene and null mutation of *ADH6* (and knockout of the aldehyde dehydrogenase-encoding gene *Hfd1*, fatty acyl-CoA synthetase genes *FAA1* and *FAA4*, and fatty acyl-CoA oxidase-encoding gene *POX1*), plus the expression of *FaCoAR* (bifunctional fatty acyl-CoA reductase), and integrating an extra copy of *MmCAR* (carboxylic acid reductase from *Mycobacterium marinum*) under the control of the Gal7 promoter and glucose limitation in a fed-batch manner, they reported the best improvement in *S. cerevisiae,* producing 1.5 g.L^−1^ of fatty alcohol. The production of fatty alcohol such as palmitoleic alcohol (C16-1) and oleic alcohol (C18-1), among others, surpassed the level of the products shown by *E. coli*, and the yeast has the advantage of being generally recognized as safe (GRAS) by the FDA. These authors developed yeast cell factories to produce fatty alcohols (e.g., oleic acid and palmitoleic acid, among others), alkanes (e.g., pentadecane), and free fatty acids. This finding is relevant because it opens the possibility of constructing yeast cell factories to produce other valuable compounds, e.g., vanillin.

Another ADH application is producing aromatic compounds, for which there is higher demand in the industry. Gargouri et al. [146] studied the use of a system of two enzymes, hydroperoxide lyase (HPLS) from plants (*Mentha spicata*, *M. piperita*) and ADHs from yeast or horse liver, to produce aldehydes or alcohols selectively, taking advantage of the fact that the addition of cofactors like NAD(P)^+^ or NAD(P)H controls the direction of the ADH enzyme. In this way, the purity of hexanal improved because the hydrolysis of 13-hydroperoxy linoleic acid was coupled to the oxidation of a small amount of hexanol. In other words, the presence of the second enzyme, ADH, supported the efficacy of the first enzyme, HPLS, so the coupled system HPLS/ADH improved the total rate conversion [146].

Recently, Zheng et al. [147] and Zhou et al. [148] published broad biotechnological applications of ADHs in the production of chiral pharmaceutical intermediates, including antidepressant, antiasthmatic, antiepileptic, and anticancer drugs. ADHs from fungi participate in the production of some of these drugs. For example, the yeasts *Rhodosporidium toruloides* and *Candida viswanathii* participate in the production of the antidepressant *S*-duloxetine. In the case of the production of (*R*)-3-hydroxy-4,4,4-trifluorobutanoate ethyl ((*R*)-EHTB), an intermediate of befloxatone—a potent antidepressant—the cells of *Saccharomyces uvarum* and *Kluyveromyces marxianus* [149,150] were used in a biphasic system for the asymmetric reduction of 3-oxo-4,4,4-trifluorobutanoate ethyl ketone (EOTB) to (*R*)-EHTB with 85% and 81% conversion, respectively, while the use of solid-phase purified Adh showed the highest conversion of >99.9% [151].

Intermediate ketone synthesis is challenging because they involve stereoselective reduction. These types of substrates contain sterically and electronically similar substitutions on both sides of the carbonyl group, making them hard to selectively convert substrates; Zhou et al. [148] widely reviewed ketones’ asymmetric reduction via ADHs or ketoreductases mainly from bacteria and fungi and the strategies to improve the stereoselectivity and efficiency of these enzymes. One example is the ADH from *Kluyveromyces polyspora*, which can perform the stereoselective reduction of diarylmethane. Alternatively, the fungus *Magnaporthe grisea*’s ADH showed stereoselectivity in reducing tetralone derivatives [148]. Thus, ADHs’ use in chiral alcohol synthesis via asymmetric reduction is one of the most valuable applications of these enzymes in the industry.

### 7.3. Participation of ADH in Fuel Cells

One of ADH´s most exciting biotechnology applications is its use in biofuel cells. These biofuel cells can transform chemical to electric energy using biocatalysts and are helpful in biodevices that operate in extreme environments like high temperatures [152]. In this sense, the high thermostability of the ADH enzyme of microorganisms capable of growing in extreme conditions increases the power density and lifetime of the biofuel cells. One example is *Sulfolobus tokodaii*; its *St*ADH was successfully used for this purpose [152]. This study opens the possibility of using ADHs such as *Hp*ADH3 from the yeast *O. polymorpha*, which displays thermostability and an increased power density of the fuel cells.

### 7.4. Use of ADH Gene Promoters for Expressing Genes of Interest in Organisms for Industrial Applications

ADH gene promoters are widely used for heterologous gene expression due to their regulatory mechanisms. For example, promoters of the *ADH1* and *ADH2* genes of *S. cerevisiae* have been used to express heterologous proteins in this yeast because the *ADH1* promoter is a strong constitutive promoter and the *ADH2* promoter is strongly regulated [153]. The regulon *alcA*/a*lcR* for ethanol utilization from *A. nidulans* has been widely used for the expression of heterologous proteins because of the strong regulation by AlcR over the promoter of *alcA* (ADHI), e.g., in fungi and microalgae like *Chlamydomonas reinhardtii* [154,155].

The yeast *R. toruloides* is essential in the industry due to its natural ability to produce value-added oleochemicals and pigments. Sun et al. [156], with the idea for further metabolic engineering of this yeast, utilized the promoter of the *RtADH2* gene (P*ADH2*). This promoter permitted the regulation of the expression of genes under its control by growing in glucose-deficient media supplemented with galactose. In this report, an inducible flippase–flippase recognition target (FLP/FRT) system was developed, where the *RtADH2* promoter controlled the *FLP* gene. *FLP* and *FRT* are involved in homologous recombination for postintegration cleavage of exogenous DNA in the oily yeast *R. toruloides*. Thus, with this regulated system *PADH2-FLP/FRT*, recycling the antibiotic marker gene to integrate multiple genes of interest is possible in this yeast used in the industry.

Ergün et al. [157] used the alcohol dehydrogenase-2 promoter (P*ADH2*) from *K. phaffii (Pichia pastoris)* to generate variations capable of up-regulation and increased expression under stringent conditions, such as ethanol catabolism, termed novel engineered promoter variants (NEPVs). Briefly, they focused on the transcription factor binding sites (TFBSs) identified via in silico analysis and by their one-for-one replacement with synthetic motifs for the Mxr1, Cat8, and Aca1 binding sites, the integration of a synthetic TATA box, and/or nucleosome optimization using GFP as a reporter. In this nucleosome optimization, systematic changes were made at each cycle in a single nucleotide in the sequences between the TFBSs, and those candidates with the lowest cumulative score were selected. They observed that several P_ADH2_ NEPVs were better than the wild type and that the P_ADH2-Cat8_ NEPV was the best and had the highest expression at 20 h of fermentation. Therefore, novel engineering to change the structure of a promoter impacting regulatory circuits increases the possibility of improving processes on an industrial scale.

### 7.5. Improvement in Qualities of ADHs: Via Immobilization or Protein Engineering

For the industrial use of ADHs from *S. cerevisiae*, one challenge is the increase in its stability against changes in temperature and pH and the reusability of the enzyme. The immobilization and stability of ADH enzymes to different solid carriers, like polyvinyl alcohol fibers, glyoxyl agarose, and nanoparticles, have been reported to overcome these problems. Using polyvinyl fibers as a carrier improved the yeast Adh1p enzyme, making it more tolerable to rough industrial conditions and maintaining excellent performance even at a pH of 9, thermostability at high temperatures, such as 60 °C to 80 °C, and resistance at up to eight reusability cycles, conserving at the end at least 60% of the original activity [158].

Ottone et al. [159] reported the immobilization of the tetrameric yeast Adh1p to glyoxyl agarose via crosslinking with polyethylenimine (PEI) to achieve the oxidation of long-chain fatty alcohol like tetracosanol to produce lignoceric acid. The Adh1p was more stable against thermal inactivation and pH variations because immobilization reduces the dissociation and aggregation of the enzyme and generates a microenvironment that makes it less sensitive to pH variations. Moreover, the immobilized Adh1p was equally efficient with the same reaction rate with substrates of different-length chains and permitted the reusability of the enzyme at least three times in batch fermentation. This Adh enzyme was used to convert CO_2_ to methanol [160]. Briefly, in a three-step sequential reaction using NADH as a cofactor in each one, the formate dehydrogenase (FDH) hydrogenates CO_2_ to produce formic acid, which in turn is reduced by formaldehyde dehydrogenase (FaldDH), producing formaldehyde; finally, the Adh reduces formaldehyde to produce methanol. So, in this case, the chemical immobilization of the yeast Adh1p to titanium nanoparticles with glutaraldehyde described by Ghannadi et al. [160] resulted in an enzyme with higher storage stability that was more stable at different pHs and temperatures; moreover, the enzyme was active (87%) even after ten recycling processes and overcame substrate inhibition in the conversion of formaldehyde to methanol, compared with the free counterpart. Therefore, the immobilized Adh1p enzyme has the advantages of stability, recyclability, and overcoming inhibition by substrate, which could be used in other industrial processes in which it participates.

The engineering of ADHs is a golden opportunity. The decrease or increase in steric hindrance via the mutagenesis of ADHs is an effective tool that can be used to modify the stereoselectivity of these enzymes [161]. Another potential ADH application is the effect of charged residues in stereoselectivity because of charged attraction/repulsion mechanisms described for carbonyl reductase [162]. The HCSM strategy (hydroclassified combinatorial saturation mutagenesis) in a *KpADH* mutant library permitted the identification of a variant with exceptionally high stereoselectivity [163]. The HCSM technique focused on the identification of potential hot spots in substrate-binding pocket residues (PocS and PocL) of the *Kp*ADH, selected for their hydrophobicity and steric hindrance. Then, via overlap extension PCR, the complete *Kp*ADH product was obtained with these simultaneously mutated hot spots at each residue group. Cloned variants result in libraries with high diversity in bulk, hydrophobicity, and substituents, increasing the probability of identifying all variants with greater than 95% enantioselectivity. In this way, they were able to select a clone with high binding affinity, catalytic efficiency, and the highest enantioselectivity of 99.4%, which has the requirements for purity of pharmaceutical intermediates [163]. These findings strongly support the idea that protein engineering could be used to modulate stereospecificity in ADH-catalyzed reactions according to industrial requirements. Therefore, immobilizing genetically redesigned enzymes in processes could further improve efficiency and yield.

Table 5 shows some of the biotechnological applications of ADHs and their promoters, developed from the knowledge generated by many research laboratories.

### 7.6. ADH-Related Patents

There are many patents related to the use of *S. cerevisiae* in the production of fermented beverages; for instance, patent US4814188A [164] describes the use of a mutant in the *ADH1* gene for a beer with a low alcohol level or without alcohol. There is an increased number of patents related to the use of *ADH* genes or their promoters in the production of bioethanol, such as patent US20130040353A1 [165], or to remove fermentation inhibitors, such as patent WO2013/178915 A1 [166].

Other patents are related to the use of *ADH* promoter sequences to produce compounds of pharmaceutical or cosmetic value, such as miniproinsulins or leptins (patents US5866371A, US6183985B1, US8222386B2 [167,168,169]); viral antigens (patent US4769238A [170]); and compounds that prevent misfolding, fibril formation, or protein aggregation that causes neurodegenerative diseases [171]. The patent US5695973A describes the use of ADH enzyme to produce a “green scent” or “fresh notes” (leaf alcohol (cis-3-hexen-1-ol) and leaf aldehyde (trans-2-hexenal)) [172].

ADH enzymes can be used to produce alkyl alcohols, such as in patent CN102762722B [173]. Some *ADH* genes have be modified to produce fatty dicarboxylic acids, such as in patent CA2841794C [174], and fuels and chemical products, such as in patent US9012189B2 [175]. Another example is patent US8859151B2, which is related to a biofuel cell with AdhA immobilized in biocathodes [176], among many others. Table 6 shows examples of ADH-related patents.

In summary, the fungal ADH enzymes and the promoters of the respective genes have wide biotechnological applications. Some examples have been mentioned, and their continuous study, highlighting the importance of ADHs and their multiple applications, opens more possibilities for improving industrial processes and innovation.

## 8. Conclusions

Alcohol dehydrogenase enzymes are present in organisms as diverse as bacteria, fungi, plants, and animals. The ADH enzymes catalyze the interconversion of alcohols, aldehydes, and ketones by reducing NAD^+^ or NADP^+^ to NADH or NADPH, respectively. Fungal ADHs play a key role in various cellular processes such as morphogenesis; the maintenance of complex biological interactions, whether they are mutualist or parasitic to plants, insects, or mammals; and energy metabolism. During energy metabolism, fungi use fermentative or oxidative ADH enzymes with a variety of substrates, such as primary alcohols like ethanol as a carbon source or aromatic alcohols like aryl alcohols, which participate in lignin degradation and protect from stress produced by certain chemical agents. Most of the fungal ADH enzymes studied belong to the family of Zn-dependent ADHs, although a few are non-Zn-dependent or iron-dependent ADHs. In general, the role of the different enzymes, as well as their transcriptional regulation, is better known in the yeast *S. cerevisiae* and in some filamentous fungi such as *A. nidulans*. Although the fundamental knowledge gained from actual studies of the transcriptional regulation and physiological function of fungal ADH enzymes has been translated into many important biotechnological applications, little is known about such enzymes in many fungi. In the past few years, many fungal genomes have been sequenced, providing information about the number of *ADH* genes contained in different fungal species. Here, we observed that the number of Zn-dependent ADHs in the genome is more related to the lifestyle than the genome size of the fungus. However, very few genes have been studied to understand under which conditions such genes are transcribed or why some fungi contain many *ADH* genes while others contain only one. Most of all, the transcriptional and post-translational regulation as well as the biochemical features and functions of the many ADH enzymes are unknown, which provides an opportunity to characterize these enzymes to gain insights into their various functions under different conditions. Thus, it is of great importance to carry out studies on the physiological roles of the different enzymes encoded in the genome of unconventional yeasts, as well as of filamentous fungi of different divisions, to increase the variety of novel applications and improve processes wherein ADH enzymes can be utilized.

## Figures and Tables

**Table 1:** Features of Zn-dependent fungal ADHs.

Organism	Enzyme	Substrate(s)	Cofactor(s)	Oligomeric State	Cellular Location	Metabolic Function	Reference
*Aspergillus* *nidulans*	ADHI	Acetaldehyde	NADH	Octameric	Cytoplasm	Ethanol production during glucose fermentation	[37]
	ADHII	Ethanol	NAD^+^	Unknown	Cytoplasm	Ethanol utilization under carbon starvation	[38]
	ADHIII	Ethanol	NAD^+^	Tetrameric	Unknown	Ethanol utilization under anaerobiosis	[39]
*Botrytis cinerea*	*Bc*ADH1	Unknown	Unknown	Unknown	Unknown	Ethanol production during glucose fermentation	[36]
*Candida maltosa*	*Cm*ADH1	Acetaldehyde	NAD^+^/H	Homo- and hetero- tetrameric	Cytoplasm	Ethanol production during glucose fermentation	[17]
	*Cm*ADH2A	Acetaldehyde	NADH	Homo- and hetero- tetrameric	Cytoplasm	Ethanol production during xylose fermentation, under oxygen limitation	
	*Cm*ADH2B	Acetaldehyde	NADH	Unknown	Unknown	Unknown	
*Candida utilis*	ADH1	Ethanol, 1-propanol, 1-butanol	NADPH	Unknown	Cytoplasm	Unknown	[18]
*Fusarium* *oxysporum* f. sp. *lycopersici*	Adh1	Ethanol	NAD^+^	Unknown	Unknown	Ethanol production during glucose fermentation/ethanol oxidation for its use as a carbon source	[35]
*Kluyveromyces lactis*	*Kl*ADHI	Ethanol	NAD^+^	Tetrameric	Cytoplasm	Ethanol production during glucose fermentation All four enzymes participate in the oxidation of ethanol for its use as a carbon source	[1,11,12]
	*Kl*ADHII	Ethanol	NAD^+^	Tetrameric	Cytoplasm		
	*Kl*ADHIII	Ethanol	NAD^+^	Tetrameric	Mitochondria		
	*Kl*ADHIV	Ethanol	NAD^+^	Tetrameric	Mitochondria		
*Metarhizium acridum*	ADH1	Acetaldehyde	NADH	Unknown	Unknown	Ethanol production during glucose fermentation under hypoxic conditions	[40]
*Metarhizium* *anisopliae*	AdhIp	Acetaldehyde	NADH	Homo- tetrameric	Cytoplasm	Ethanol production during glucose fermentation	[41]
*Mucor lusitanicus*	ADH1	Ethanol	NAD^+^	Homo- tetrameric	Cytoplasm	Ethanol production during glucose fermentation/ethanol oxidation for its use as a carbon source	[33,34]
*Neurospora crassa*	ADH1	Primary alcohols	NADP^+^	Unknown	Cytoplasm	Ethanol production during glucose fermentation	[24]
	ADH3	Unknown	Unknown	Unknown	Cytoplasm	Ethanol oxidation for its use as a carbon source	
*Phanerochaete chrysosporium*	Aryl alcohol dehydrogenase (*Pc*Aad1p)	Aryl- and linear aldehydes	NAD^+^/ NADPH/	Monomeric	Cytoplasm	Lignin degradation pathway Response to chemical stress	[42,43]
*Saccharomyces cerevisiae*	Adh1p	Acetaldehyde	NAD^+^/ NADH	Homo- tetrameric	Cytoplasm	Ethanol production during glucose fermentation	[44]
	Adh2p	Ethanol	NAD^+^/ NADH	Homo- tetrameric	Cytoplasm	Ethanol oxidation for its use as a carbon source/ethanol production during glucose fermentation, in the absence of Adh1p	[4]
	Adh3p	Ethanol	NAD^+^/ NADH	Homo-tetrameric	Mitochondria	Ethanol oxidation for its use as a carbon source/ethanol production during glucose fermentation, in the absence of Adh1p	
	Adh4p	Ethanol	NAD^+^/ NADH	Homodimeric	Mitochondria	Ethanol oxidation for its use as a carbon source	
	Adh5p	Ethanol	NAD^+^/ NADH	Unknown	Cytoplasm	Ethanol oxidation for its use as a carbon source	
	Adh6p	Cinnamyl alcohol	NADPH	Hetero-dimeric	Unknown	Degradation of lignin	
	Adh7p	Cinnamyl alcohol	NADPH	Homodimeric	Unknown	Degradation of lignin	
*Scheffersomyces stipitis*	*Ss*ADH1	Acetaldehyde/ethanol	NAD^+^/ NADH	Unknown	Cytoplasm	Ethanol production during xylose fermentation, under oxygen limitation/ Both enzymes participate in ethanol oxidation for its use as a carbon source	[16]
	*Ss*ADH2	Ethanol	NAD^+^	Unknown	Cytoplasm		

**Table 2:** Factors involved in *ADH* gene regulation.

Organism	Gene	Inductor	Repressor	Reference
*Aspergillus nidulans*	*alcA*	Ethanol	Glucose	[75]
	*alcB*		Glucose	[38]
	*alcC*	Anaerobic stress		[39]
*Aspergillus fumigatus*	*alcC*	Anaerobic stress in infected mice		[87]
*Candida albicans*	*ADH1*	Glucose; constitutive	Constitutive	[72,73]
	*ADH2*	Growth phase		[73]
*Candida maltose*	*ADH1*	Glucose		[17]
	*ADH2A*	Ethanol		[17]
	*ADH2*B	N.D.	N.D.	[17]
*Candida utilis*	*ADH1*		*ADH1* antisense	[88]
*Fusarium oxysporum*	*Adh1*	Hypoxic conditions		[35]
*Kluyveromyces lactis*	*ADH1*	Glucose	Ethanol	[69]
	*ADH2*	Glucose	Ethanol	[69]
	*ADH3*		Ethanol	[70,71]
	*ADH4*	Ethanol		[70,71]
*Metarhizium acridum*	*Adh1*	Hypoxic conditions		[40]
*Metarhizium anisopliae*	*Adh1*	Induced during *Plutella xylostella* infection		[41]
*Mucor lusitanicus*	*Adh1*	Glucose		[33]
*Saccharomyces cerevisiae*	*ADH1*	Glucose	Ethanol Zinc deficiency	[53] [56]
	*ADH2*	Ethanol	Glucose Acetate	[57,58] [61]
	*ADH3*		Glucose	[62,63]
	*ADH4*	Zinc deficiency Lithium	DMSO	[64] [65] [67]
	*ADH5*	DMSO		[67]
*Scheffersomyces stipites*	*ADH1*	Oxygen limitation		[80]
	*ADH2*		Ethanol, glycerol	[80]

**Table 3:** Roles of ADHs in biological interactions.

Biological Interaction	Organism	Function	Reference
Parasitic	*F. oxysporum* f. sp. *lycopercici*	Adh1 positively regulates virulence during tomato-plant infection.	[35]
Parasitic	*M. anisopliae*	*adh1* mRNA is accumulated during insect-infection process and positively regulates virulence.	[41]
Parasitic	*A. fumigatus*	*alcC* mRNA is accumulated during hypoxic growth. Although it has a positive role in fungal burden in the lungs, it was not relevant to mice death.	[87]
Parasitic	*B. cinerea*	Adh1 positively regulates development, adaptation to stress, and hypoxia and virulence.	[36]
Parasitic	*A. baumannii*	Adh4 plays a key role in ethanol metabolism, while Adh3 and Adh6 were involved in the oxidative stress response and virulence.	[96]
Parasitic	*M. lusitanicus*	Adh1 negatively regulates virulence in mice. Its absence increased the fungal burden.	[81]
Parasitic	*C. albicans*	Adh1 negatively regulates biofilm formation and positively regulates virulence in mice.	[86] [92]
Parasitic	*S. sclerotiorum*	*Ss*CAD regulates sclerotia formation but does not have an effect on virulence.	[95]
Mutualistic	*Azoarcus* sp. strain *BH72*	ExaA2 positively regulates the bacterial ability to colonize the roots of rice and fix nitrogen.	[97]
Mutualistic	*Cyberlindnera americana*	Increases in the expression of aryl-alcohol dehydrogenase genes that enable the resistance to high amounts of toxic terpenes inside beetles during the colonization of pine trees.	[98]
Mutualistic	*Ambrosiella and Raffaelea*	Enables the use of ethanol-rich substrate to grow against unwanted competing fungi.	[99]

**Table 4:** Zinc-dependent alcohol dehydrogenase (Zn-ADH) in the fungal kingdom.

Phylum	Subphylum	Class	Species	Strains	Genome (Mb)	MDR	Zn-ADH
Basidiomycota	Pucciniomycotina	Pucciniomycetes	*Puccinia graminis*	CRL 75-36-700-3	88.72	4	2
	Ustilaginomycotina	Ustilaginomycetes	*Ustilago maydis*	521	19.66	13	5
	Agaricomycotina	Agaricomycetes	*Lactarius pseudohatsudake Armillaria ostoyae Laccaria bicolor*	88 C18/9 S238N-H82	99.70 60.10 64.87	4 16 5	1 7 2
		Dacrymycetes	*Calocera cornea*	HHB12733	33.24	13	5
		Tremellomycetes	*Cryptococcus neoformans*	JEC21	19.05	15	6
		Wallemiomycetes	*Wallemia mellicola*	CBS 633.66	9.8	7	3
Ascomycota	Pezizomycotina	Pezizomycetes	*Tuber indicum*	Tubin_1	110.49	7	2
		Orbiliomycetes	*Dactylellina haptotyla (Monacrosporium haptotylum)*	CBS 200.50	39.53	9	4
		Eurotiomycetes	*Aspergillus nidulans*	FGSC A4	30.27	30	11
		Dothideomycetes	*Alternaria rosae*	BMP 2777	32.84	18	10
		Lecanoromycetes	*Lasallia pustulata (Umbilicaria pustulata)*	A1-1	32.91	10	5
		Leotiomycetes	*Botrytis cinerea*	B05.10	42.63	13	8
		Sordariomycetes	*Metarhizium robertsii Trichoderma reesei Neurospora crassa Fusarium oxysporum f. sp. lycopersici*	ARSEF 23 QM6a OR74A 4287	41.65 33.39 41.10 53.91	19 17 14 49	8 11 7 30
		Xylonomycetes	*Xylona heveae*	TC161	24.33	11	6
	Saccharomycotina	Saccharomycetes	*Saccharomyces cerevisiae* *Candida albicans* *Kluyveromyces lactis* *Komagataella phaffii (Pichia pastoris)*	S288C SC5314 CBS 2105 GS115	12.15 14.28 10.90 9.21	9 10 14 6	7 4 12 5
	Taphrinomycotina	Schizosaccharomycetes	*Schizosaccharomyces pombe*	972h-	12.59	3	1
Mucoromycota	Glomeromycotina	Glomeromycetes	*Gigaspora margarita*	BEG34	773.1	24	6
	Mortierellomycotina	Mortierellomycetes	*Linnemannia elongata (Mortierella elongata)*	AG-77	49.85	6	2
	Mucoromycotina	Mucoromycetes	*Mucor lusitanicus*	1006PhL	36.34	11	6
Zoopagomycota	Zoopagomycotina	Zoopagomycetes	*Piptocephalis cylindrospora*	RSA 2659	10.74	3	1
	Entomophthoromycotina	Basidiobolomycetes	*Basidiobolus meristosporus*	CBS 931.73	89.48	30	25
	Kickxellomycotina	Dimargaritomycetes	*Dimargaris cristalligena*	RSA 468	30.65	5	4
Blastocladiomycota		Blastocladiomycetes	*Catenaria anguillulae*	PL171	41.33	3	1
Chytridiomycota		Chytridiomycetes	*Caulochytrium protostelioides*	ATCC 52028	10.62	4	2
		Monoblepharidomycetes	*Gonapodya prolifera*	JEL478	48.79	11	2
		Neocallimastigomycetes	*Piromyces finnis Neocallimastix* sp.	fin sp3	56.45 200.96	1 4	1 3
Microsporidia			*Mitosporidium daphniae*	UGP3	5.6	1	1
Cryptomycota			*Rozella allomycis*	CSF55	13.44	2	1

Taxonomy information of the fungal kingdom was obtained from MycoCosm and NCBI Taxonomy (https://mycocosm.jgi.doe.gov/mycocosm/home (accessed on 30 June 2023) and https://www.ncbi.nlm.nih.gov/taxonomy/ (accessed on 30 June 2023)). The numbers of proteins in the fungal genomes of MDR and ADH-Zn were obtained by performing blastp at NCBI (https://blast.ncbi.nlm.nih.gov/Blast.cgi?PROGRAM=blastp&PAGE_TYPE=BlastSearch&LINK_LOC=blasthome (accessed on 30 June 2023)) using the ADH1 protein from *Saccharomyces cerevisiae* (ID: CAA99098).

**Table 5:** ADHs’ biotechnological applications.

Biofuels Production			
Target	Organism	Bioprocess	Reference
*Hp*ADH3	*Ogataea polymorpha*	Ethanolic fermentation	[141]
ADHIII	*Neurospora crassa*	Ethanolic fermentation	[22]
ADHIV, ADHV	*Scheffersomyces stipites*	Detoxifying aldehyde inhibitors	[143]
Pharmaceutical and cosmetic industries			
Target	Organism	Bioprocess	Reference
Adh1p	*Saccharomyces cerevisiae*	Aromatic compounds	[146]
Adh1p	*Saccharomyces cerevisiae*	Antidepressant	[147]
Adh5p, Adh6p	*Saccharomyces cerevisiae*	Fatty alcohol synthesis	[145]
*Kp*ADH	*Kluyveromyces polyspora*	Chiral alcohol synthesis	[148]
ADH promoters			
Promoter	Organism	Bioprocess	Reference
P*alcA*	*Aspergillus nidulans*	System for alcohol-inducible gene expression	[155]
P*ADH2*	*Rhodosporidium toruloides*	System pADH2-FLP/FRT for recycling antibiotic marker gene	[156]
P*ADH2*	*Pichia pastoris* (*Komagataella phaffii*)	Up-regulation by variants for transcription factor binding sites	[157]
Improvement of qualities			
Target	Organism	Strategy	Reference
Adh1p	*Saccharomyces cerevisiae*	Immobilization	[158,159,160]
Adh1p	*Saccharomyces cerevisiae*	Polarity scanning and combinatorial mutagenesis	[161]
*Kp*ADH	*Kluyveromyces polyspora*	Hydroclassified saturation and combinatorial mutagenesis	[163]

**Table 6:** Examples of ADH-related patents.

Product	Subject of Patenting or Patented Invention	Patent No.	Reference
Bioethanol from a pentose substrate	*Saccharomyces cerevisiae*-modified strain with mutated *SsXp* gene up-regulated by *Sc*P*_ADH_* promoter and genes involved in pentose fermentation.	US20130040353A1	[165]
Bioethanol from xylose	*S. cerevisiae* capable of fermenting xylose, expressing the xylanase gene (Xl) up-regulated by *Sc*P*_ADH_*, and resistant to acetic acid.	WO2013/178915 A1	[166]
Fuel and chemical products	*S. cerevisiae* strain with an ADH with high catalytic efficiency, reduction, or elimination of 3-keto-acid and/or aldehyde derivatives byproducts.	US9012189B2	[175]
Lower alkyl alcohol (butanol)	Modified *ADH6* or *ADH7*, among other *ADH* genes, for butanol production in *S. cerevisiae* or other microorganisms.	CN102762722B	[173]
Fatty dicarboxylic acids	*Candida* spp. with ADH2a increased activity.	CA2841794C	[174]
Low-alcohol or alcohol-free beer	Yeast mutant in the *ADH1* gene.	US4814188A	[164]
Miniproinsulin, leptins, or derivatives thereof	Yeast mutants with up-regulated genes by yeast *ADH2* promoter.	US5866371A US6183985B1	[167] [168]
HBsAg antiviral antigen	Yeast over-expressing S-protein from Hepatitis B virus up-regulated by the *Sc*P*_ADH1_* promoter.	US4769238A	[170]
Green scents: leaf alcohol (cis-3-hexen-1-ol) and leaf aldehyde (trans-2-hexenal)	*Geotrichum candidum* strain which expresses an ADH which converts leaf aldehydes to leaf alcohols.	US5695973A	[172]

## Data Availability

The data presented in this study are available inside this article.
